# Detection of Highly Pathogenic Avian Influenza A(H5N1) Virus in Cat and Rats during Outbreak in Backyard Poultry, United States, 2025

**DOI:** 10.3201/eid3210.260418

**Published:** 2026-10

**Authors:** Suzanna M. Storms, Jade Rathmann, Lynette Hemker, Miranda Vieson, Leyi Wang

**Affiliations:** Texas A&M Veterinary Medical Diagnostic Laboratory, College Station, Texas, USA (S.M. Storms); University of Illinois College of Veterinary Medicine, Urbana, Illinois, USA (S.M. Storms, M. Vieson, L. Wang); Greenville Veterinary Clinic, Ltd, Greenville, Illinois, USA (J. Rathmann, L. Hemker)

**Keywords:** Highly pathogenic avian influenza virus, influenza, viruses, respiratory infections, zoonoses, H5N1, cat, rat, poultry, United States

## Abstract

In 2025, highly pathogenic avian influenza A(H5N1) virus was detected in a poultry flock in Illinois, USA. Quantitative reverse transcription PCR, sequencing, and histopathology on cat and rat samples from the farm showed multiple positive tissues and high sequence identity to an avian isolate. Small mammals might contribute to H5N1 transmission.

The current panzootic of highly pathogenic avian influenza (HPAI) A(H5N1) clade 2.3.4.4b virus emerged in Europe in 2020 and by 2022 had spread throughout Europe, Africa, and North America (*1*–*4*). Trans-Atlantic introduction into North America was first documented in December 2021 and has since persisted in wild and domestic birds, has been detected in dairy cattle, and has been reported sporadically in other mammals (*5*). Waterfowl have played an equal role to seabirds in virus spread, which is concerning given their higher likelihood of overlapping ranges with poultry farms (*2*,*6*). We describe an HPAI H5N1 outbreak in a backyard poultry flock in Illinois, USA, with subsequent spillover into domestic and wild mammals on the premises.

## The Study

On March 12, 2025, a farmer in southern Illinois with a small backyard poultry flock brought remains of 6 animals to their primary veterinarian for a necropsy workup after acute illness and near-complete mortality (100/105 birds) of the flock. The farm, ≈2 miles from a 25,000-acre reservoir lake within the Mississippi Flyway, housed 4 turkeys (*Meleagris gallopavo*), 1 goose (*Anser* sp.), 20 Guinea fowl (*Numida* spp.), 80 chickens (*Gallus gallus domesticus*), 17 cattle, 15 cats, and 2 dogs. Because HPAI virus (HPAIV) was suspected, the 6 animals, a chicken, a turkey, a goose, 2 Guinea fowl, and a rat (*Rattus norvegicus*), were sent to the University of Missouri Veterinary Medical Diagnostic Laboratory (VMDL; Columbia, MO, USA) for postmortem examination. VMDL collected and evaluated fresh and formalin-fixed tissues and performed influenza A virus (IAV) quantitative reverse transcription PCR (qRT-PCR) on 1 chicken sample, according to guidelines from the US Department of Agriculture (USDA) National Animal Health Laboratory Network (NAHLN; https://www.aphis.usda.gov/labs/nahln). The sample tested presumptive positive per NAHLN guidelines, and VMDL subtyped it as H5 influenza. VMDL then forwarded the sample to the National Veterinary Services Laboratories (NVSL; Ames, IA, USA) for confirmatory testing, which verified HPAIV H5N1 clade 2.3.4.4b virus. 

Further PCR testing of the avian species for *Mycoplasma gallisepticum*, *M. synoviae*, and avian paramyxovirus were all negative. The necropsy findings reported hepatitis, pulmonary hemorrhage, and pulmonary edema in the poultry species and bronchopneumonia and meningoencephalitis in the rat. No additional testing was performed on the rat or other bird species. Upon report of HPAIV on the farm, state and federal agencies were notified. The Illinois Department of Agriculture and USDA quarantined the farm and monitored it for 4 months.

On March 16, 2025, the primary veterinarian collected the remains of a 13-year-old domestic shorthaired cat and a second feral rat from the farm. In conjunction with Illinois Department of Agriculture and USDA, the cat and rat specimens were sent to the University of Illinois Veterinary Diagnostic Laboratory (VDL; Urbana, IL, USA) on March 19. VDL performed gross necropsy and collected fresh and formalin-fixed tissues. VDL screened lung tissues from the cat and rat for IAV by qRT-PCR, following NAHLN guidelines. The cat lung tested presumptive positive (cycle threshold [Ct] value 21.2), subtyped as H5; NVSL confirmed H5N1 clade 2.3.4.4b virus. The rat lung tissue was negative upon initial screening, but NVSL conducted follow-up testing on tracheal swab samples, which were H5N1-positive.

In addition, we performed follow-up IAV qRT-PCR testing on 12 tissue samples from the cat and 15 tissue samples from the rat and considered Ct values <45 IAV-positive. All 12 cat tissue samples were IAV-positive (Ct values 10.9–31.1), and 11/15 rat tissues were IAV-positive (Ct 17.1–39.4) (Figure 1). Brain tissues of both animals had the highest viral loads (cat Ct 10.9; rat Ct 17.1). Of note, tracheal and nasal swab samples from both animals were IAV-positive (Ct range 22.1–29.5). A second lung sample from the rat returned a Ct of 39.4. Kidney tissues from both species were IAV-positive (cat Ct 20.9; rat Ct 34.6), and urine from the rat also tested IAV-positive (Ct 32.0). Nucleic acid in feces from the cat had a Ct value of 28.6, but no nucleic acid was detected in rat feces.

**Figure 1:**
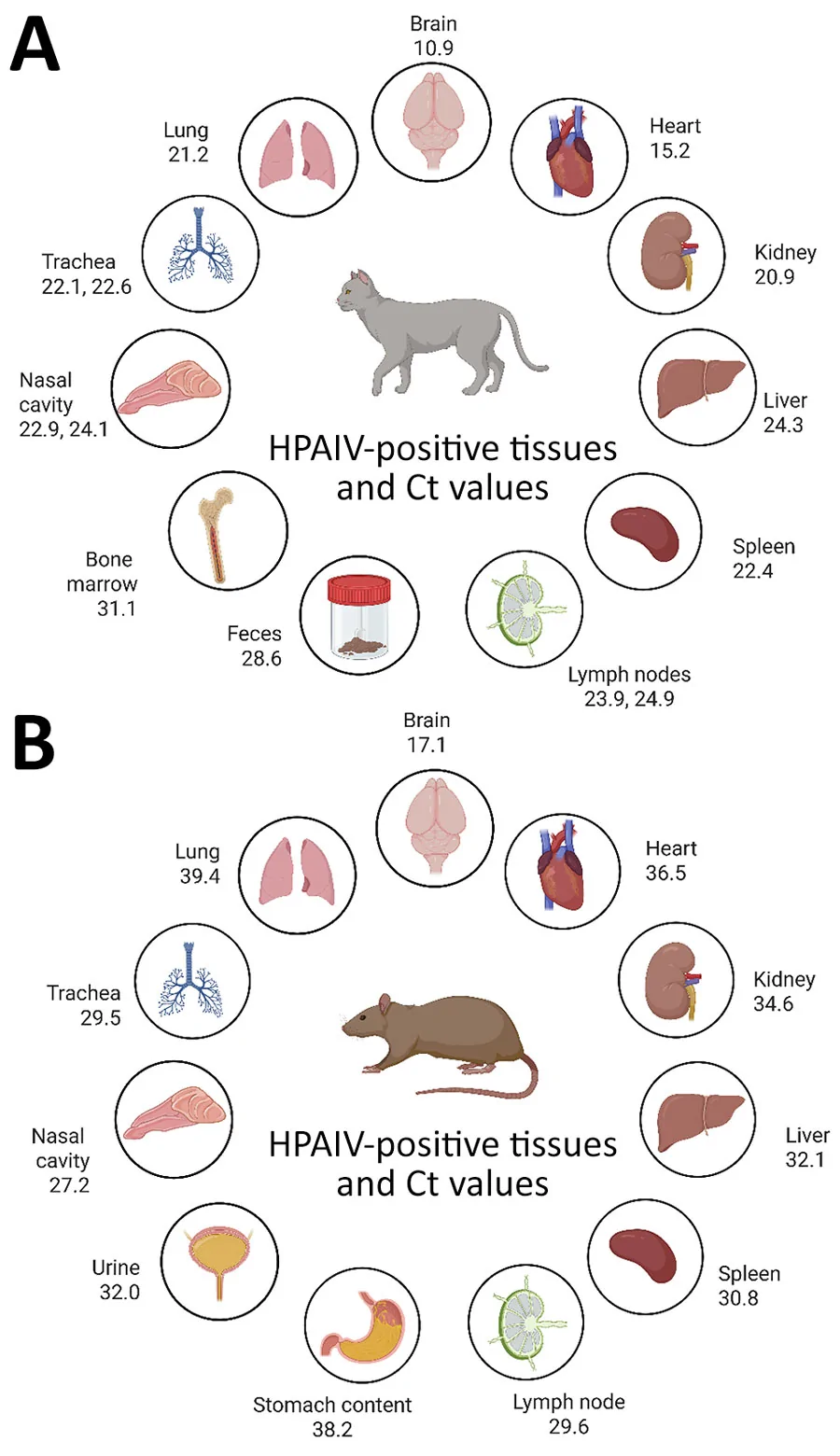
Quantitative reverse transcription PCR results from HPAIV A(H5N1) in cat and rats during outbreak in backyard poultry, United States, 2025. Specimens obtained from a cat (A) and a rat (B) found dead on farm where the outbreak occurred were confirmed to be positive for H5N1 clade 2.3.4.4b, genotype D1.1 virus. Graphic shows HPAIV-positive tissues and corresponding Ct values. A) For the cat, Ct values are shown for mesenteric and peripheral lymph nodes; urine, stomach content, and small intestine were not tested. B) For the rat, Ct values for peripheral lymph node only are shown; no mesenteric lymph node was tested. Also, rat feces, bone marrow, small intestine, and testis were tested, but HPAIV was not detected in those organs. Figure created with BioRender (https://www.biorender.com). Ct, cycle threshold; HPAIV, highly pathogenic avian influenza virus.

We performed whole-genome sequencing on 3 samples from the cat (brain, heart, and nasal swab) and 1 brain sample from the rat by using an amplicon-based enrichment protocol and sequencing on a MiSeq platform (Illumina, https://www.illumina.com). We used the Galaxy platform (*7*) for sequence assembly. All samples returned complete sequences for 8 segments (GenBank accession nos. PX687012–27) (Appendix Table). In addition, we included sequence data of the HPAIV H5N1 strain 25-009367-001 (GenBank accession nos. PV616546–53) from the chicken sample submitted by VMDL to NVSL in the analysis. Phylogenetic tree analysis revealed that the chicken, rat, and cat strains clustered together with other avian and mammal HPAIV (Figure 2; Appendix Figures 1–8). All 3 animal species from the outbreak contained the polymerase basic 2 E627K mutation that is associated with mammalian adaptation (*8*). All cat samples shared identical consensus sequences. We found 2 amino acid mutations, neuraminidase G392D and nucleoprotein K372E, in the cat and rat samples but not in the IAV strain from the chicken. In addition, we noted 1 synonymous polymerase basic 2 C1552T mutation in the rat strain that we did not see in cat and chicken strains. The genotype of the outbreak virus was D1.1 (*9*) (Appendix).

**Figure 2:**
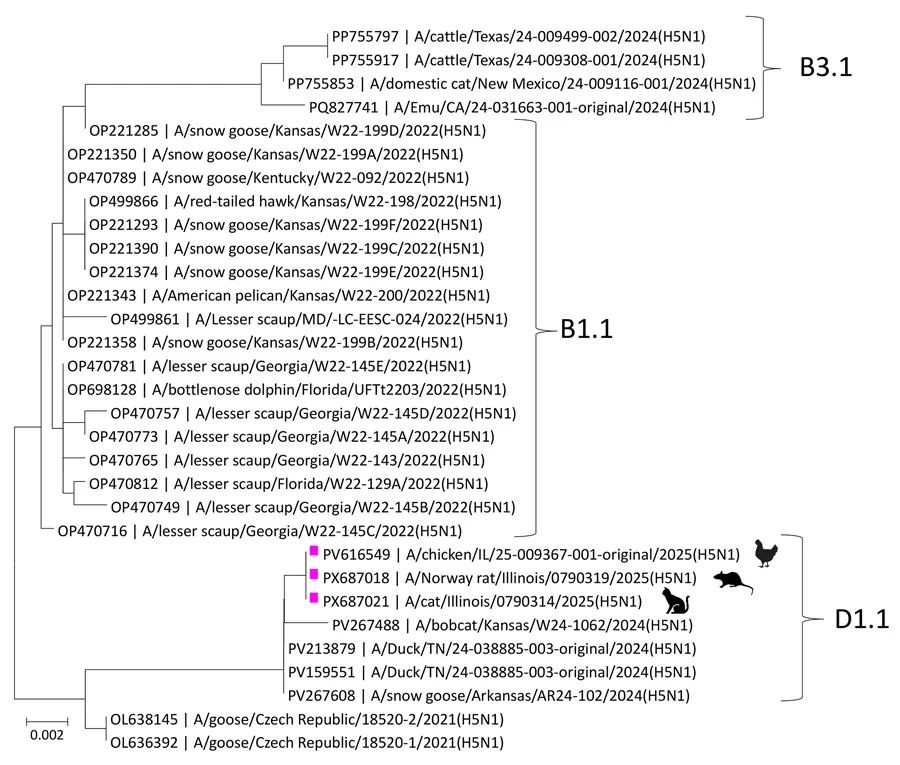
Phylogenetic tree of hemagglutinin gene sequences from highly pathogenic avian influenza A(H5N1) virus in cat and rats during outbreak in backyard poultry, United States, 2025. The cat, rat, and chicken from this study (pink squares) are closely related to other avian strains, as well as an isolate from a bobcat (GenBank accession no. PV267488). GenBank accession numbers are provided. Scale bar indicates nucleotide substitutions per site.

Gross examination during necropsy showed multifocal red mottling of the cat and rat lungs and additional right cranioventral lung consolidation in the rat. Histopathology findings in the cat included multifocal bronchointerstitial pneumonia, mild multifocal nonsuppurative meningoencephalitis, and acute adrenocortical necrosis (Figure 3, panels A–C). We noted severe cranioventral bronchopneumonia (not shown) amongst a background of diffuse alveolitis in the rat lung tissue (Figure 3, panel D), likely related to a bacterial infection. Additional lesions in the rat possibly related to viral infection included mild nonsuppurative periventricular encephalitis in brain tissue (Figure 3, panel E) and myocarditis. Immunohistochemistry for IAV in the cat lung showed apical airway epithelial immunoreactivity (Figure 3, panel A, inset), and brain tissue showed multifocal glial and neuronal immunoreactivity (Figure 3, panel C). We also noted mild periventricular immunoreactivity in neurons in the rat brain (Figure 3, panel F).

**Figure 3:**
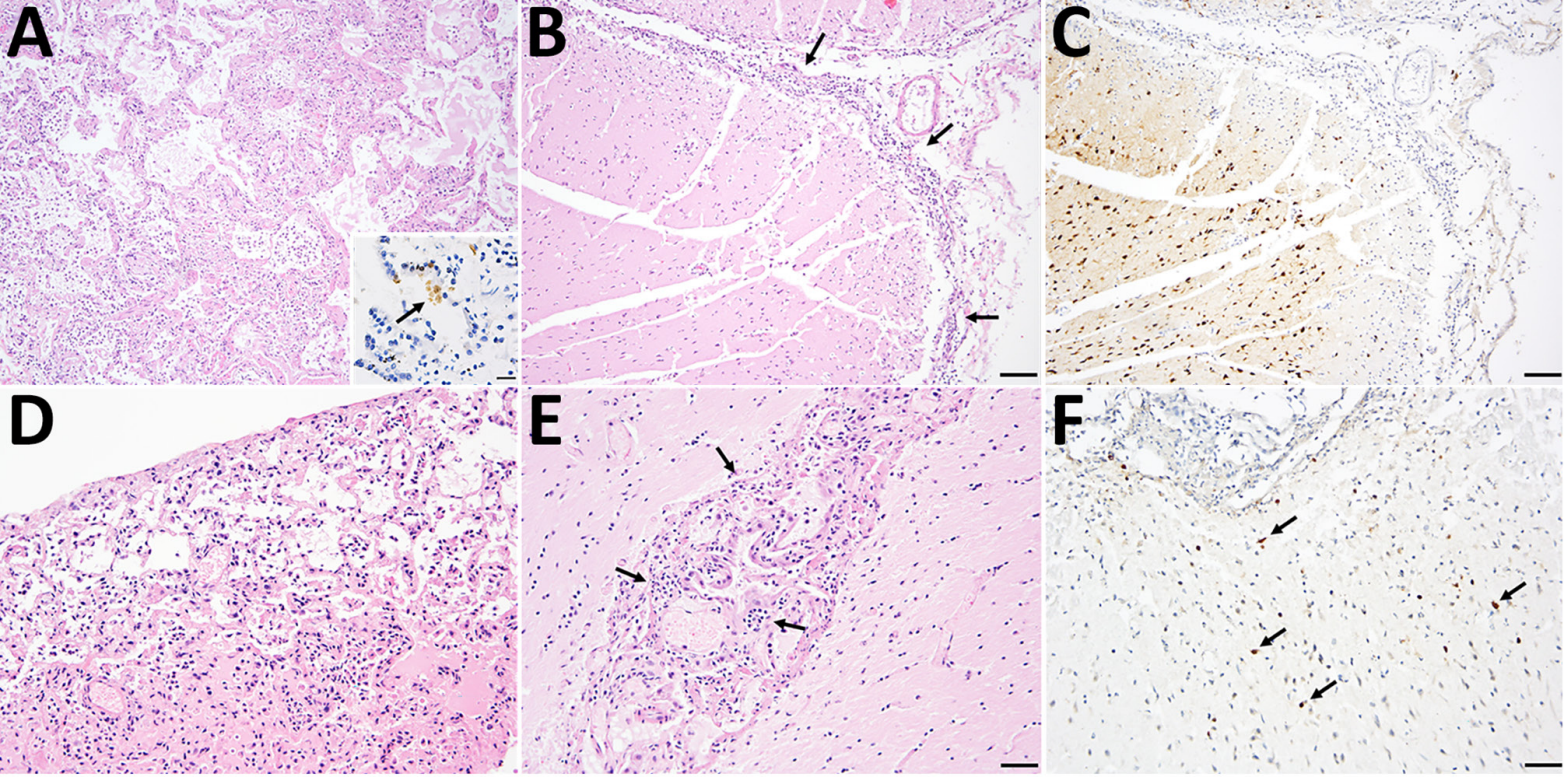
Histopathology and immunohistochemistry of lesions in a cat and rat found dead during outbreak of highly pathogenic avian influenza A(H5N1) virus in backyard poultry, United States, 2025. A–C) Cat samples; D–F) rat samples. A) Hematoxylin and eosin (H&E) stain of feline lung tissue showing bronchointerstitial pneumonia; original magnification ×10. Inset (immunohistochemical [IHC] stain) shows positive immunoreactivity (arrow) of the cytoplasm of apical epithelial cells within distal airways. Scale bar indicates 20 μm. B) H&E stain of feline brain tissue showing nonsuppurative meningoencephalitis (arrows). Scale bar indicates 100 μm. C) IHC stain of feline brain showing strong immunoreactivity in cerebrocortical glial cells and neurons. Scale bar indicates 100 μm. D) H&E stain of rat lung tissue showing background alveolitis. Original magnification ×20. E) H&E stain of rat brain tissue showing nonsuppurative periventricular encephalitis (arrows). Scale bar indicates 50 μm. F) IHC of rat brain tissue showing influenza immunoreactivity in few periventricular neurons (arrows). Scale bar indicates 50 μm.

## Conclusions

We describe a strain of H5N1 that resulted in high mortality in a poultry flock and fatal illness in small mammals on a backyard farm in Illinois, USA. Our results show that extensive systemic H5N1 virus spread occurred in both cat and rat tissues after natural infection. Rat nasal turbinates and trachea preferentially expressed α2,3-linked sialic acid receptors relative to lung, likely explaining the lower Ct values in upper respiratory tissues and presence of bacterial bronchopneumonia, potentially leading to increased viral shedding (*10*).

Cat feces and urine have previously been shown to harbor H5N1 virus (*11*,*12*), which our results corroborate. Rats have previously been reported to harbor HPAIV in agricultural settings and have been detected in the current epizootic (*13*,*14*). Detection of HPAIV in rat urine and kidney tissue suggests that rodent urine could be a potential transmission medium and warrants further investigation. Rats reside on many farms, can predate birds, and might serve as an intermediate step between infected avian species and susceptible mammalian hosts (*15*). That coexistence is particularly threatening for commercial poultry and swine confinement farms, which frequently harbor rodent populations despite strict biosecurity efforts. A limitation of our study was that no virus isolation was performed on tissues or urine due to biosafety constraints.

Although cats have been shown to participate in HPAIV transmission, our findings indicate that further investigation is needed to clarify the role of rodents. This study raises awareness of small mammals at the wildlife–livestock interface and highlights the potential role of rats in influenza transmission to commercial livestock production.

Appendix Additional information on detection of highly pathogenic avian influenza A(H5N1) virus in cat and rats during outbreak in backyard poultry, United States, 2025.
