# Supplementary material for: Detection of Highly Pathogenic Avian Influenza A(H5N1) Virus in Cat and Rats during Outbreak in Backyard Poultry, United States, 2025
Source: Emerg Infect Dis. 2026 Oct;32(10):1721–5. doi: 10.3201/eid3210.260418 (PMC13632871; doi:10.3201/eid3210.260418)
Supplement: Supplementary file 1 [file 26-0418-Techapp-s1.pdf]

Article DOI: https://doi.org/10.3201/eid3201.260418

EID cannot ensure accessibility for supplementary materials supplied by authors. Readers who have difficulty accessing supplementary content should contact the authors for assistance.

Detection of Highly Pathogenic Avian Influenza A(H5N1) Virus in Cat and Rats during Outbreak in Backyard Poultry, United States, 2025

Appendix

Materials and Methods

Initial influenza A virus (IAV) RT-qPCR screening and reporting of lung tissue of the cat and the rat were performed according to NAHLN protocol using standard operating procedures. After initial screening, we performed RT-qPCR on fresh tissues with modified extraction and amplification protocols to determine where virus was present throughout the tissues. Appendix Table displays the RT-qPCR results for the 12 cat and 15 rat tissues that were tested.

Tissue homogenization was performed using 10% tissue to PBS ratio in bead beater tubes. Fecal samples were processed in a 10% feces to PBS ratio, and vortexed for three minutes. After processing, samples were centrifuged for 3 minutes at 6,000 × g, and 200 µL of sample supernatant was used for extraction.

RNA extraction was performed using the MagMax Pathogen RNA/DNA kit (Applied Biosystems, Foster City, California, USA). For each extraction well, the following kit components were added: 20µl mixture of 10 µL lysis enhancer and 10 µL of magnetic beads, 200 µL sample, 400 µL lysis mixture of 200 µL lysis/binding solution, 2 µL carrier RNA, and 200 µL isopropanol.

The RT-qPCR protocol, per reaction is: 1 µL probe, 1 µL reverse primer1, 1 µL reverse primer2, 1 µL forward primer, 1 µL Xeno internal amplification control, 1 µL LIZ for Xeno, 6.25 µL TaqPath 1-Step Multiplex Master Mix (cat. A28523, Applied Biosystems), 4.75 µL

molecular grade water, 8 µL extracted RNA, for a total reaction volume of 25 µL. A positive (matrix) and negative control (nuclease free water) were used for each run in place of RNA extract. The thermocycler protocol is as follows: Reverse transcription at 50°C for 15 minutes, polymerase activation at 95°C for two minutes, and 45 cycles of 95°C for 15 seconds and 60°C for 60 seconds. We ran the RT-qPCR on a BioRad thermocycler, and analyzed the data with the CFX Maestro (version 1.0) software and considered reactions with cycle threshold (Ct) <40.0 to be positive.

Whole-genome sequencing

WGS was performed on −80°C stored RNA that were extracted as described above. A pre-enrichment protocol targeting each influenza segment was performed before sequencing from previously published primers, as described by Storms et. al (*1*). The SuperScript III One-Step RT-PCR System with Platinum Taq High Fidelity DNA Polymerase (Invitrogen, Waltham, MA, USA) was used according to the manufacturer's instructions. Briefly, 25 µL of the 2x reaction mix, 1 µL of the primer mix, 1 µL of the Superscript III RT/Taq enzyme, and 3 µL of RNase/DNase free water were used in each reaction and loaded into MicroAmp (Applied Biosystems, Waltham, MA, USA) tube strips. Twenty µL of freshly extracted RNA was added to each reaction tube.

The thermocycler program was as follows: reverse transcription at 42°C for one hour, followed by RT inactivation/initial denaturation at 94°C for two minutes. The first PCR cycle, amplifying the HA, NP, NA, and M segments, was as follows: 94°C for 30 seconds, 45°C for 30 seconds, 68°C for 7 minutes, for 20 cycles. The second PCR step, amplifying the PB2, PB1, PA, and NS genes, was as follows: 94°C for 30 seconds, 57°C for 30 seconds, and 68°C for 7 minutes for 20 cycles. The final elongation was at 68°C for 5 minutes, followed by a hold step at 4°C until the tubes were removed from the thermocycler. DNA purification was performed using the QIAquick PCR purification (Qiagen, Venlo, Netherlands) according to the kit instructions.

A Qubit dsDNA Quantification Assay kit (ThermoFisher Scientific, Waltham, MA, USA) was used to quantify DNA and dilute samples to the correct concentration for the library preparation. The library preparation was performed using the Nextera XT DNA Library Preparation Kit per Illumina's Nextera XT DNA library prep reference guide. A total amount of 1 ng of purified PCR product was used for the next-generation sequencing. Library preparation and

NGS on the Illumina MiSeq platform were conducted by the University of Illinois Veterinary Diagnostic Laboratory.

Consensus sequence assembly

FASTQ data obtained from the MiSeq instrument were processed on the EU Galaxy server (usegalaxy.eu), following the Avian influenza viral strain analysis from the gene segment sequencing data training material (*2*,*3*). Paired end FASTQ files from each sample were processed by fastp for initial pre-processing with sequences lengths <30 discarded (*4*). Reads with a mean quality below 30 were cut from the 5′ and the 3′ reads. Next, the processed FASTQ files were run with VAPOR, a tool for influenza classification for short read data, which identifies references to be used for mapping and assembly from a customizable database of 50 references (*5*). The Kmer filtering threshold was set to 0.1, and the minimum Kmer proportion was set to 0.0. The top scoring matches for each segment were used for mapping using BWA-MEM for each gene segment, using default settings (*6*). Samtools view was used to filter mapped reads to keep only those with quality scores above 20, and were paired reads with proper pairs (*7*). Mapping statistics were generated using QualiMap BamQC (*8*,*9*). Next the BAM files were split by the references selected earlier, using BamTools (*10*). The consensus sequence was generated per segment using the iVar consensus tool (*11*). The minimum quality score threshold to count base was set at 20, the minimum frequency threshold at 0.7, the minimum indel frequency threshold at 0.8, and the minimum depth to call consensus was set at 10 for the first pass. For sequences that had ambiguous bases, the frequency threshold was decreased to 0.5, with a minimum depth to call consensus set at 2. The complete workflow can be found at: https://training.galaxyproject.org/training-material/topics/variant-analysis/tutorials/aiv-analysis/tutorial.html.

Assembled gene segments of each strain were deposited into GenBank and their accessions can be found in the Appendix Table. In Appendix Table 2 are displayed the closest related strains by sequence identity per segment.

Sequence analysis

Following consensus sequence generation, the sequences of each segment were aligned using MAFFT (*12*). Aligned sequences were translated into amino acids in MEGA (version 11)

(*13*). and non-synonymous mutations were looked for and recorded. The N2 numbering system was used to standardize the numbering of substitutions (*14*).

Over 4,000 sequences for each gene segment were obtained from BV-BRC.org using the following parameters: Orthomyxoviridae, complete genome, segment, collection year (2017–2025), isolation country (USA), host group (Avian, nonhuman mammal, sea mammal). Gene segments were aligned using MAFFT via the Galaxy Europe server, followed by tree construction with MEGA (version 7.0.26). Tree files were then down sampled using PARNAS to preserve genetic diversity, but facilitate visualization (*15*). The assembled segments from the cat, rat, and GenBank sequence data of the chicken strain 25-009367-001 were then added to the downsampled data, and MEGA was used to produce a bootstrap consensus tree (1000 iterations). Phylogenetic trees were visualized and annotated by clade, subtype, and internal gene cassette using MEGA (version 7.0.26), and displayed in Figure 2 in the dispatch.

In addition, NCBI virus H5N* database was used to create phylogenetic trees for each segment. A randomized subset of all records was used, with 20 or fewer sequences stratified by host. This resulted in 1807 randomized sequences for each segment. Segments were aligned using MAFFT, followed by downsampling using PARNAS for visualization, as described above. The cat, rat, and chicken sequences were then added to the trees, and assembled and visualized as stated above. A total of 8 trees, demonstrating diversity of H5N* from year 1959 onward are displayed in Appendix Figures 1–8.

Immunohistochemistry

Tissues from both the cat and rat were collected, fixed in neutral buffered formalin, trimmed, embedded in paraffin. The processed formalin-fixed tissues were then stained with hematoxylin/eosin for histologic evaluation. For immunohistochemistry (IHC), slides were treated with 37°C 0.1% Protease solution heated in a 40°C oven for 20 minutes (Millipore Sigma, cat.no. P5147). After the pre-treatment, slides were blocked with Peroxidazed 1 for five minutes, followed by a secondary block with Background Punisher for ten minutes (Biocare Medical, Pacheco, CA, cat.nos. PX968, BP974). Slides were then incubated with the primary monoclonal antibody to influenza A virus at a 1:50 for 2 hours (Meridian Bioscience, Cincinnati, OH, cat. no. C65331M). Slides were then incubated with a secondary ani-mouse antibody for 30 minutes, followed by intelliPATH FLX DAB chromogen for five minutes and counterstained

with CAT Hematoxylin for five minutes (Biocare Medical, cat.nos. IPK5010, CATHE). All tissues were prepared by the University of Illinois Veterinary Diagnostic Laboratory.

References

1. Storms SM, Leonardi-Cattolica A, Prezioso T, Varga C, Wang L, Lowe J. Influenza A virus shedding and reinfection during the post-weaning period in swine: longitudinal study of two nurseries. Front Vet Sci. 2024;11:1482225. PubMed https://doi.org/10.3389/fvets.2024.1482225

2. Hiltemann S, Rasche H, Gladman S, Hotz HR, Larivière D, Blankenberg D, et al.; Galaxy Training Network. Galaxy Training: A powerful framework for teaching! PLoS Comput Biol. 2023;19:e1010752. PubMed https://doi.org/10.1371/journal.pcbi.1010752

3. Galaxy Community. The Galaxy platform for accessible, reproducible and collaborative biomedical analyses: 2022 update. Nucleic Acids Res. 2022;50:W345–51. PubMed https://doi.org/10.1093/nar/gkac247

4. Chen S, Zhou Y, Chen Y, Gu J. fastp: an ultra-fast all-in-one FASTQ preprocessor. Bioinformatics. 2018;34:i884–90. PubMed https://doi.org/10.1093/bioinformatics/bty560

5. Southgate JA, Bull MJ, Brown CM, Watkins J, Corden S, Southgate B, et al. Influenza classification from short reads with VAPOR facilitates robust mapping pipelines and zoonotic strain detection for routine surveillance applications. Bioinformatics. 2020;36:1681–8. PubMed https://doi.org/10.1093/bioinformatics/btz814

6. Li H, Durbin R. Fast and accurate short read alignment with Burrows-Wheeler transform. Bioinformatics. 2009;25:1754–60. PubMed https://doi.org/10.1093/bioinformatics/btp324

7. Danecek P, Bonfield JK, Liddle J, Marshall J, Ohan V, Pollard MO, et al. Twelve years of SAMtools and BCFtools. Gigascience. 2021;10:1061. PubMed https://doi.org/10.1093/gigascience/giab008

8. García-Alcalde F, Okonechnikov K, Carbonell J, Cruz LM, Götz S, Tarazona S, et al. Qualimap: evaluating next-generation sequencing alignment data. Bioinformatics. 2012;28:2678–9. PubMed https://doi.org/10.1093/bioinformatics/bts503

9. Okonechnikov K, Conesa A, García-Alcalde F. Qualimap 2: advanced multi-sample quality control for high-throughput sequencing data. Bioinformatics. 2016;32:292–4. PubMed https://doi.org/10.1093/bioinformatics/btv566

10. Barnett DW, Garrison EK, Quinlan AR, Strömberg MP, Marth GT. BamTools: a C++ API and toolkit for analyzing and managing BAM files. Bioinformatics. 2011;27:1691–2. PubMed https://doi.org/10.1093/bioinformatics/btr174

11. Grubaugh ND, Gangavarapu K, Quick J, Matteson NL, De Jesus JG, Main BJ, et al. An amplicon-based sequencing framework for accurately measuring intrahost virus diversity using PrimalSeq and iVar. Genome Biol. 2019;20:8. PubMed https://doi.org/10.1186/s13059-018-1618-7

12. Katoh K, Rozewicki J, Yamada KD. MAFFT online service: multiple sequence alignment, interactive sequence choice and visualization. Brief Bioinform. 2019;20:1160–6. PubMed https://doi.org/10.1093/bib/bbx108

13. Tamura K, Stecher G, Kumar S. MEGA11: Molecular Evolutionary Genetics Analysis Version 11. Mol Biol Evol. 2021;38:3022–7. PubMed https://doi.org/10.1093/molbev/msab120

14. World Health Organization. Summary of neuraminidase (NA) amino acid substitutions associated with reduced inhibition by neuraminidase inhibitors (NAIs) [cited 2026 Apr 27]. https://www.who.int/publications/m/item/summary-of-neuraminidase-(na)-amino-acid-substitutions-associated-with-reduced-inhibition-by-neuraminidase-inhibitors-(nais)

15. Markin A, Wagle S, Grover S, Vincent Baker AL, Eulenstein O, Anderson TK. PARNAS: objectively selecting the most representative taxa on a phylogeny. Syst Biol. 2023;72:1052–63. PubMed https://doi.org/10.1093/sysbio/syad028

Appendix Table. GenBank accession information for the complete gene segments from the cat and the rat

Sequence ID	Segment	GenBank accession no.
Rat		
IL25_0319_b.PA	PA	PX687012
IL25_0319_b.PB1	PB1	PX687013
IL25_0319_b.NP	NP	PX687014
IL25_0319_b.MP	MP	PX687015
IL25_0319_b.NA	NA	PX687016
IL25_0319_b.PB2	PB2	PX687017
IL25_0319_b.HA	HA	PX687018
IL25_0319_b.NS	NS	PX687019
Cat		
IL25_0314_b.PB1	PB1	PX687020
IL25_0314_b.HA	HA	PX687021
IL25_0314_b.NP	NP	PX687022
IL25_0314_b.NS	NS	PX687023
IL25_0314_b.PB2	PB2	PX687024
IL25_0314_b.MP	MP	PX687025
IL25_0314_b.PA	PA	PX687026
IL25_0314_b.NA	NA	PX687027

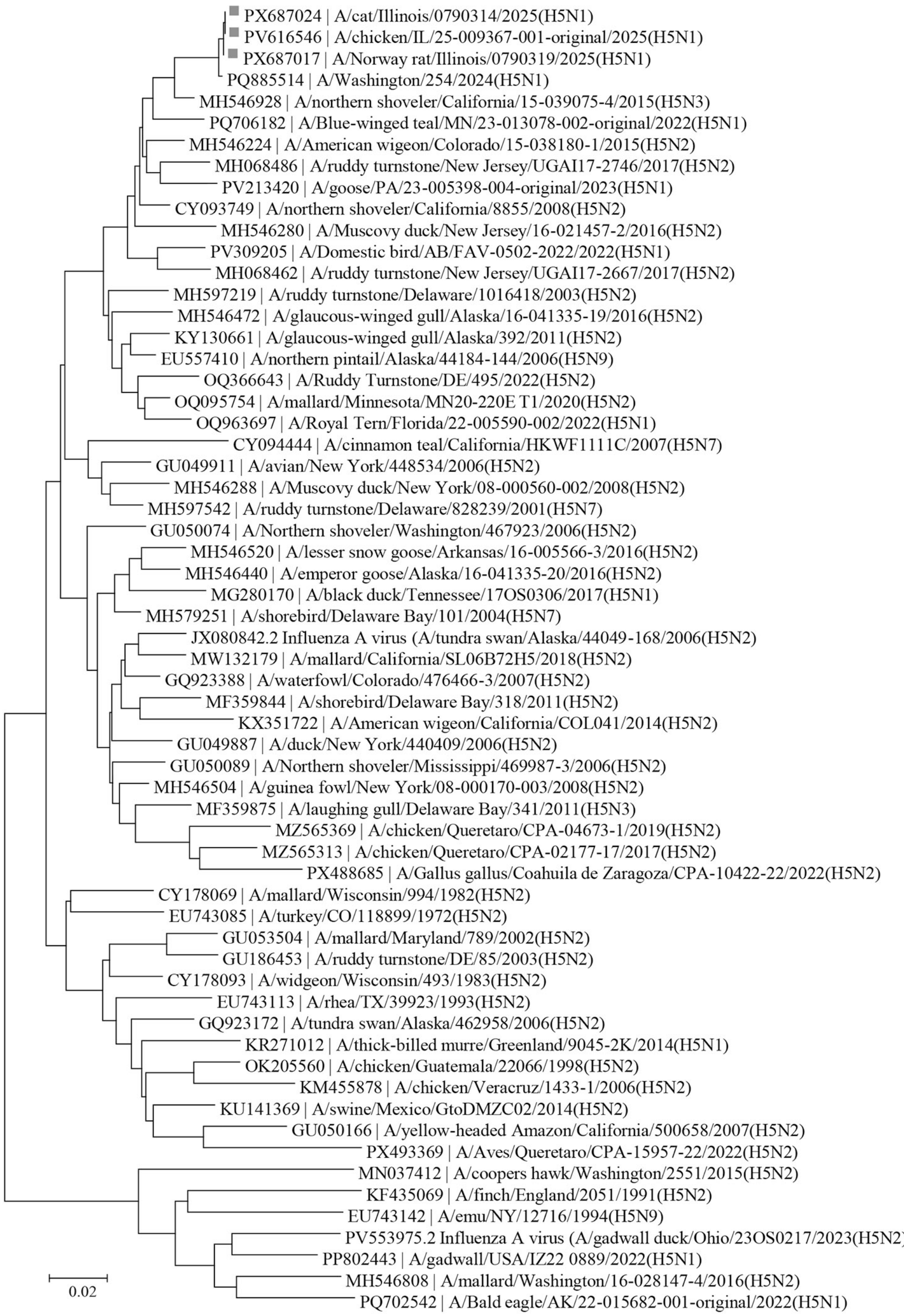

Appendix Figure 1. Phylogenetic trees of a randomized subset of H5N* PB2 genes, stratified by host, 2025. Cat, rat, and chicken strains reported in this study labeled by solid squares.

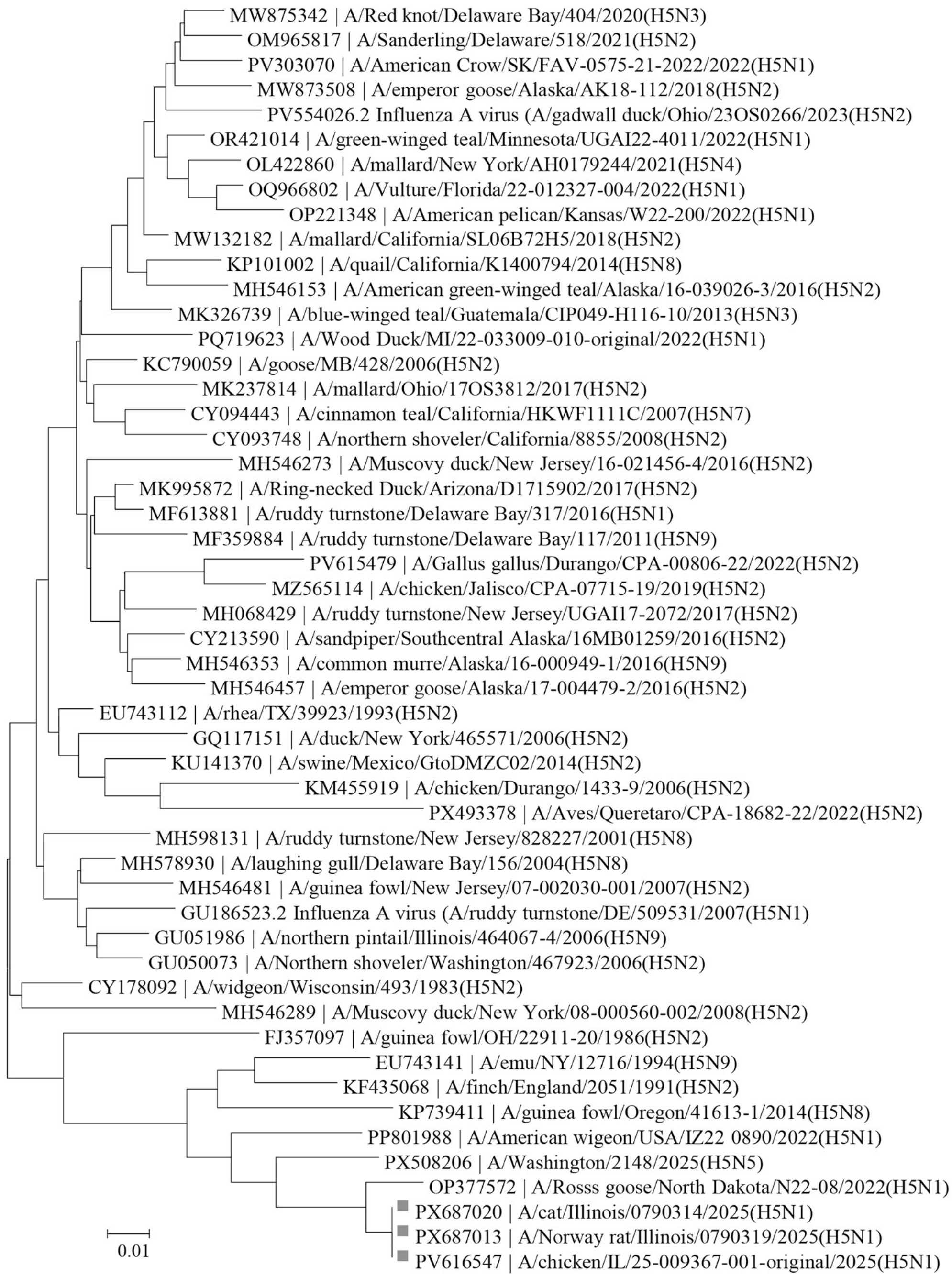

Appendix Figure 2. Phylogenetic trees of a randomized subset of H5N* PB1 genes, stratified by host, 2025. Cat, rat, and chicken strains reported in this study labeled by solid squares.

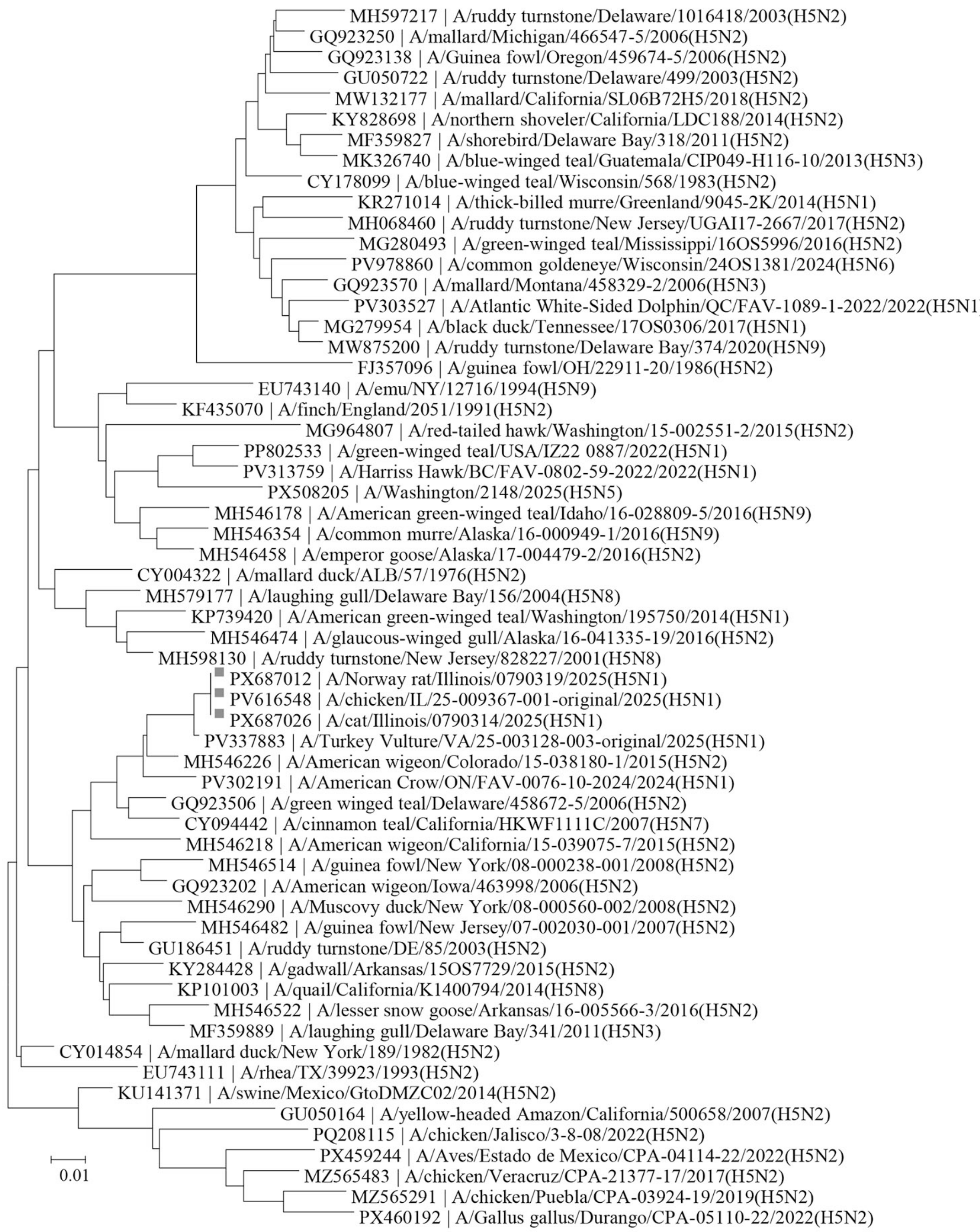

Appendix Figure 3. Phylogenetic trees of a randomized subset of H5N* PA genes, stratified by host, 2025. Cat, rat, and chicken strains reported in this study labeled by solid squares.

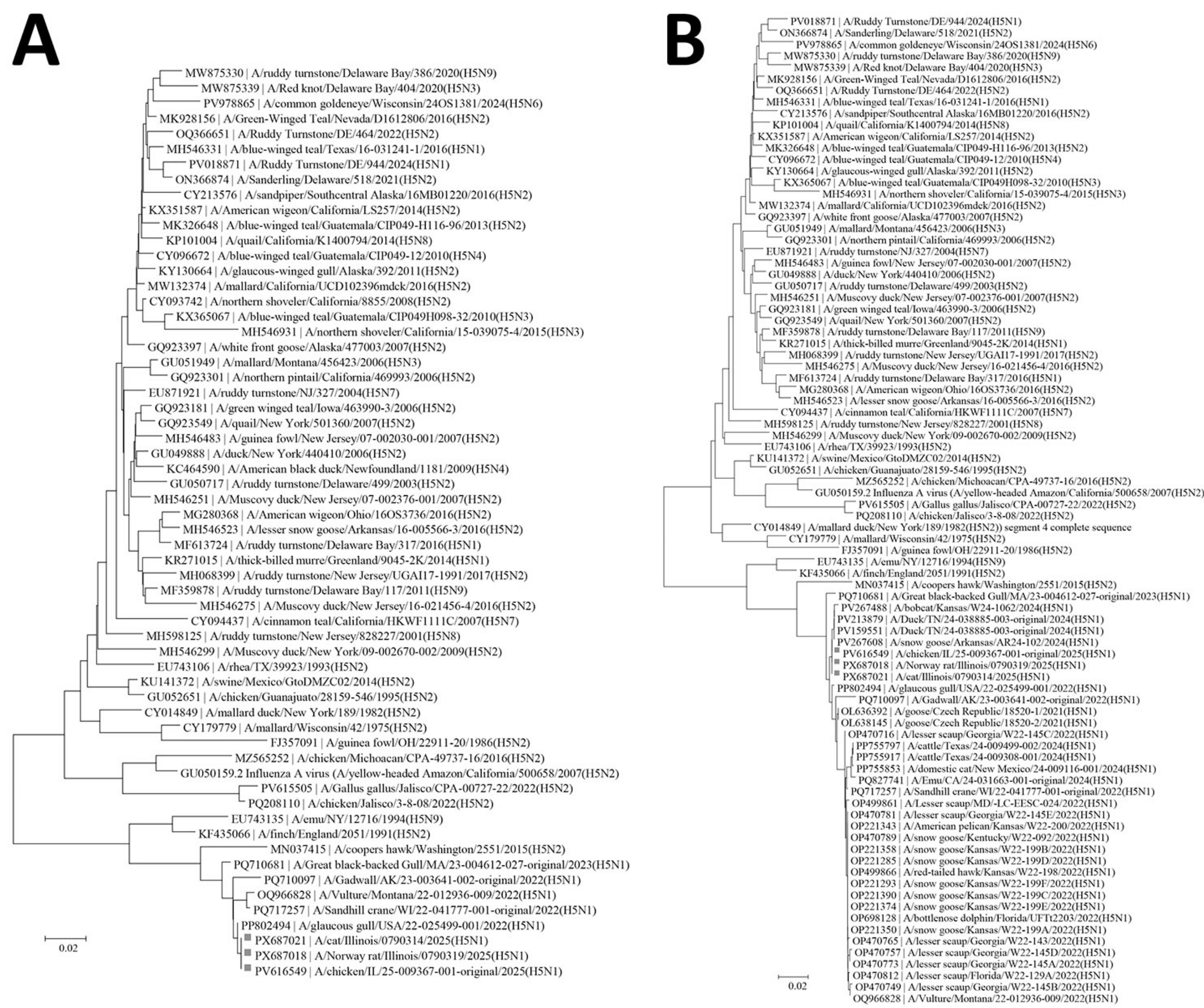

Appendix Figure 4. Phylogenetic HA gene tree analysis of HPAIV strains including cat, rat, and chicken strains reported in this study, 2025. Phylogenetic trees of a randomized subset of H5N* genes, stratified by host are displayed in the left tree. The right tree displays sequences included in main Figure 2 and all sequences in the left tree. Cat, rat, and chicken strains reported in this study labeled by solid squares.

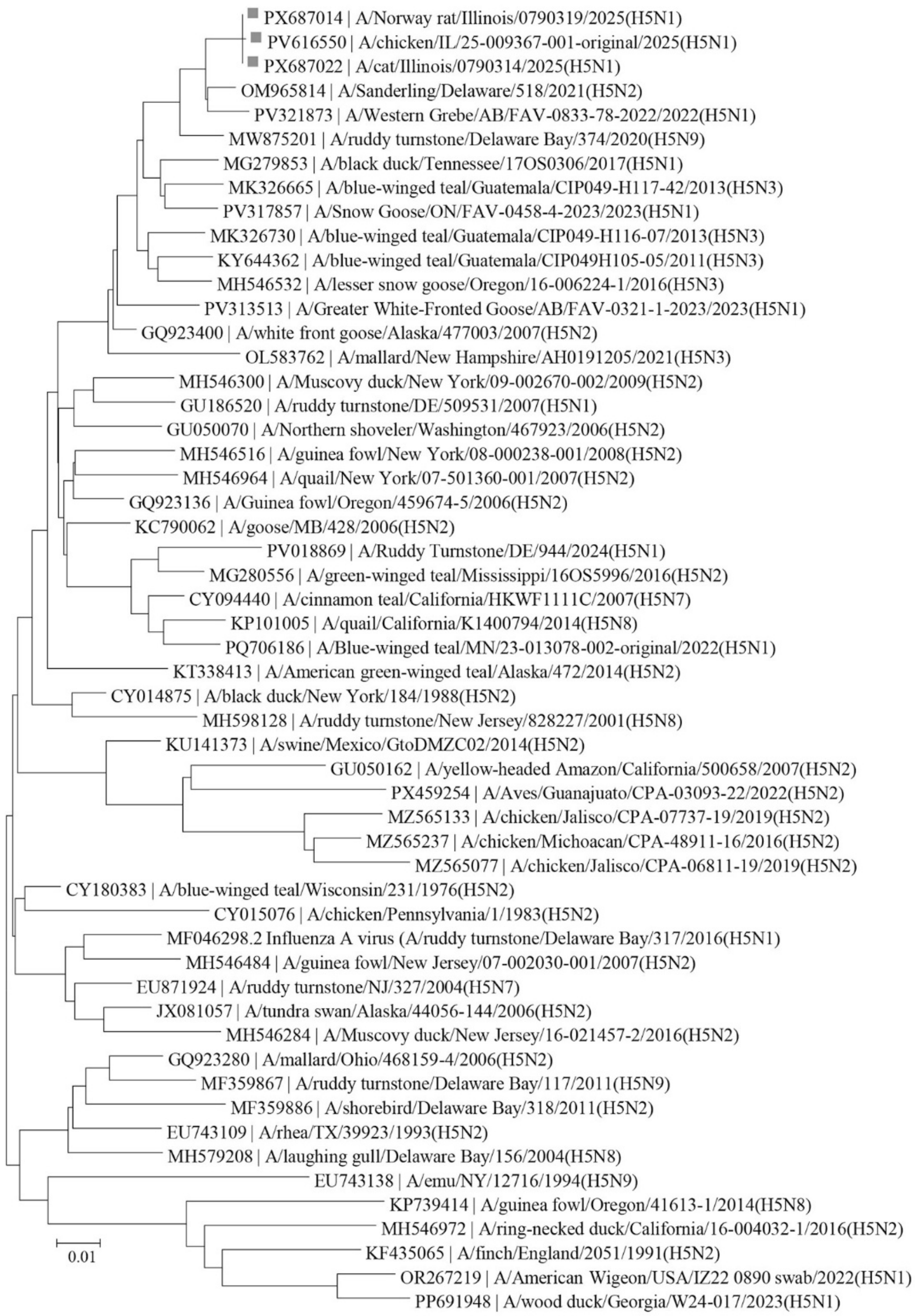

Appendix Figure 5. Phylogenetic trees of a randomized subset of H5N* NP genes, stratified by host, 2025. Cat, rat, and chicken strains reported in this study labeled by solid squares.

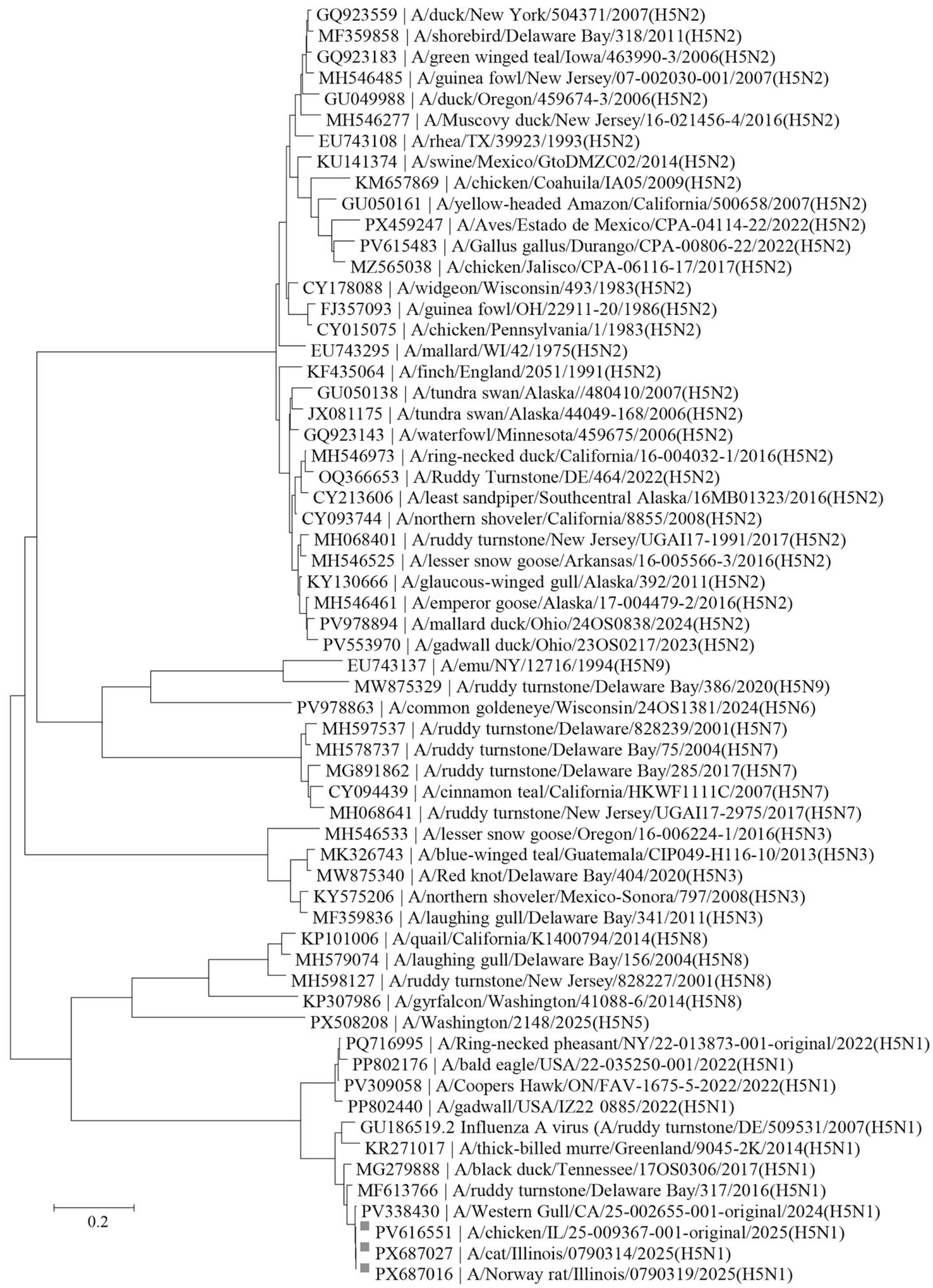

Appendix Figure 6. Phylogenetic trees of a randomized subset of H5N* NA genes, stratified by host, 2025. Cat, rat, and chicken strains reported in this study labeled by solid squares. This tree contains a diversity of neuraminidase genes, including N1, N2, N3, N5, N6, N7, N8, and N9, demonstrating the diversity of the NA gene in HPAIV.

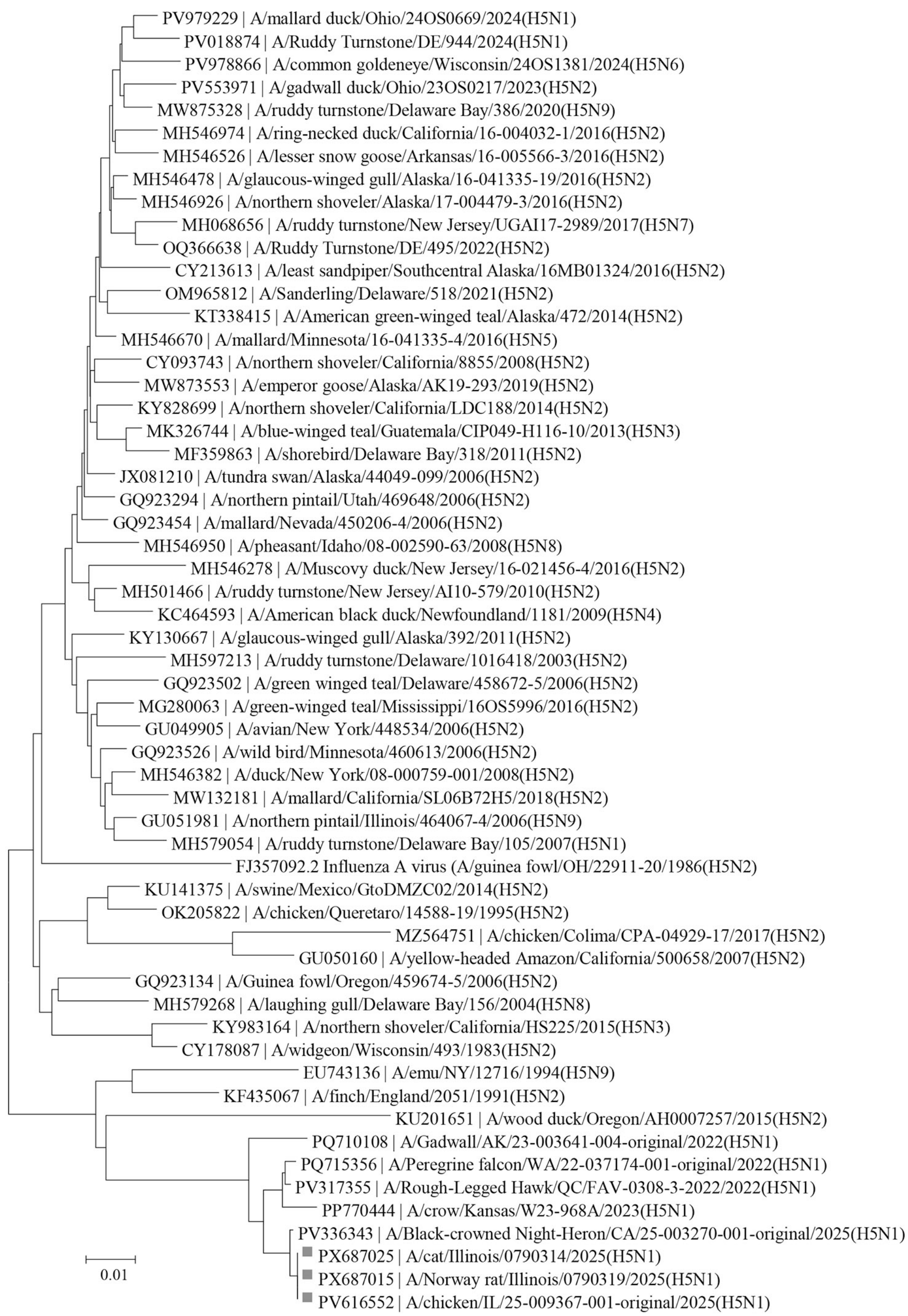

Appendix Figure 7. Phylogenetic trees of a randomized subset of H5N* M genes, stratified by host, 2025. Cat, rat, and chicken strains reported in this study labeled by solid squares.

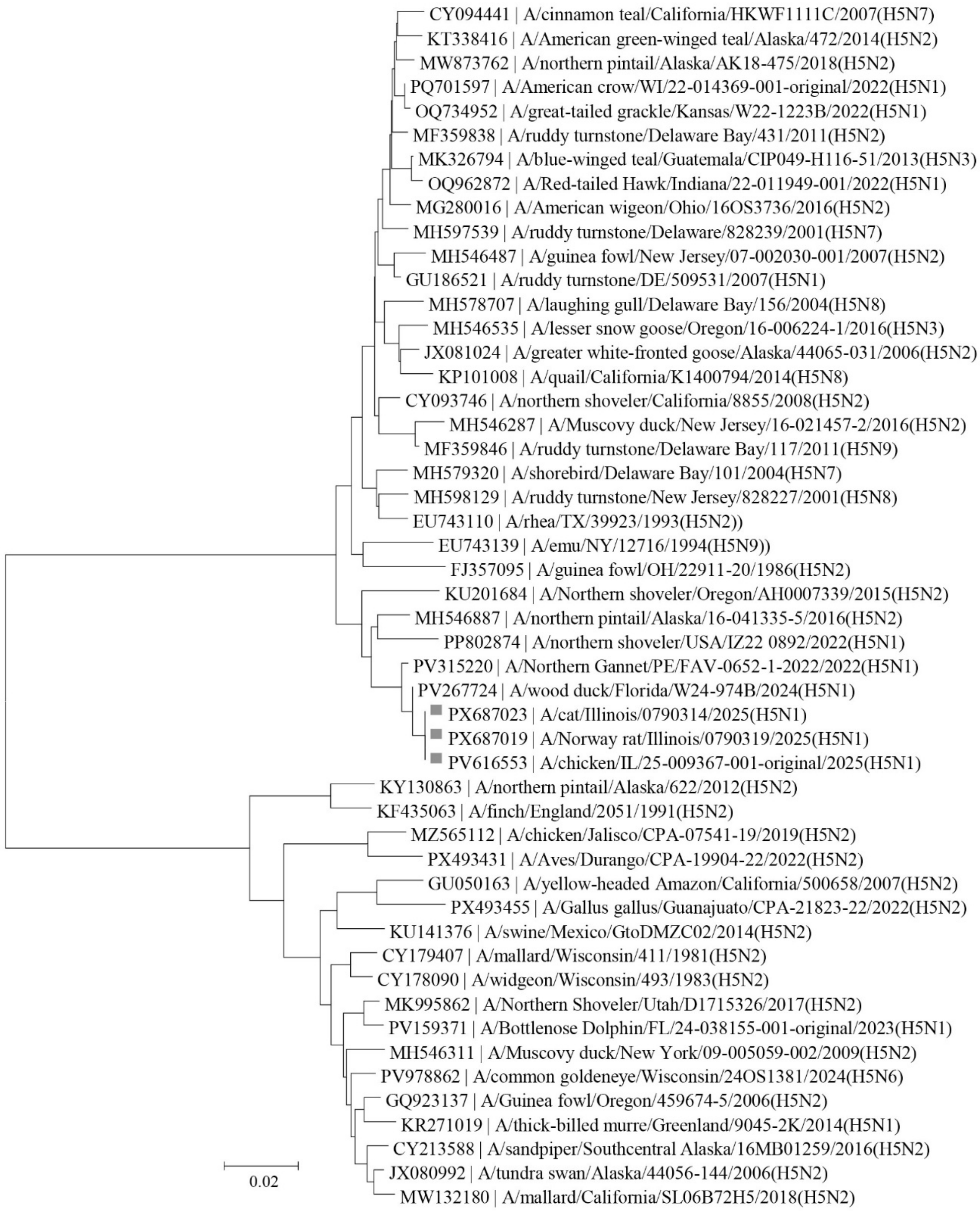

Appendix Figure 8. Phylogenetic trees of a randomized subset of H5N* NS genes, stratified by host, 2025. Cat, rat, and chicken strains reported in this study labeled by solid squares.
